# Natural and Engineered Guide RNA–Directed Transposition with CRISPR-Associated Tn7-Like Transposons

**DOI:** 10.1146/annurev-biochem-030122-041908

**Published:** 2024-07-02

**Authors:** Shan-Chi Hsieh, Joseph E. Peters

**Affiliations:** Department of Microbiology, Cornell University, Ithaca, New York, USA;

**Keywords:** gene editing, genetic engineering, transposition, horizontal gene transfer, target-site selection

## Abstract

CRISPR–Cas (clustered regularly interspaced short palindromic repeats–CRISPR-associated nuclease) defense systems have been naturally coopted for guide RNA–directed transposition on multiple occasions. In all cases, cooption occurred with diverse elements related to the bacterial transposon Tn7. Tn7 tightly controls transposition; the transposase is activated only when special targets are recognized by dedicated target-site selection proteins. Tn7 and the Tn7-like elements that coopted CRISPR–Cas systems evolved complementary targeting pathways: one that recognizes a highly conserved site in the chromosome and a second pathway that targets mobile plasmids capable of cell-to-cell transfer. Tn7 and Tn7-like elements deliver a single integration into the site they recognize and also control the orientation of the integration event, providing future potential for use as programmable gene-integration tools. Early work has shown that guide RNA–directed transposition systems can be adapted to diverse hosts, even within microbial communities, suggesting great potential for engineering these systems as powerful gene-editing tools.

## 1. INTRODUCTION

Since their discovery, CRISPR–Cas (clustered regularly interspaced short palindromic repeats–CRISPR-associated nuclease) systems have gained great attention for their programmability potential in genome editing, with promising applications in bioengineering and therapeutics (1). Standard CRISPR-mediated genome editing with the Cas9 protein generally begins with RNA-guided sequence-specific DNA cleavage, followed by host-mediated DNA repair to introduce a new DNA sequence. However, DNA double-strand breaks (DSBs) can induce detrimental stress responses and other kinds of collateral damage. Homologous recombination (HR), the DSB repair pathway that is needed to insert the new DNA sequence, must compete with nonhomologous end joining (NHEJ), which otherwise results in unwanted outcomes. Consequently, numerous strategies have been developed to avoid DSBs being formed in the editing process (2). For instance, prime editing was created to substitute a short stretch of target DNA with only nicking, and base editors were developed to potentially enable single-nucleotide changes without breaking any DNA strands. Moreover, prime editing and base editors allow only small changes and not the integration of long DNA sequences. Attempts have been made to develop DSB-free targeted integration of large cargo using engineered transposase–Cas fusions, but these efforts suffer from low efficiency and high frequencies of off-site events (3, 4). In recent years, many naturally occurring RNA-guided transposons have been discovered and characterized that have generally come to be called CRISPR-associated transposons (CASTs). These naturally evolved elements have many desirable features that hint at their promising potential for new applications in genome editing. This review focuses on how CAST elements evolved in different ways from diverse Tn7-like transposons that coopted multiple different types of CRISPR–Cas systems. Details about how these diverse systems function will help optimize their activity and bridge the gap toward the aspirational goal of designing these systems de novo.

## 2. CRISPR-ASSOCIATED TRANSPOSONS ARE TN7-LIKE TRANSPOSONS

### 2.1. Transposon Tn7

**Figure 1:**
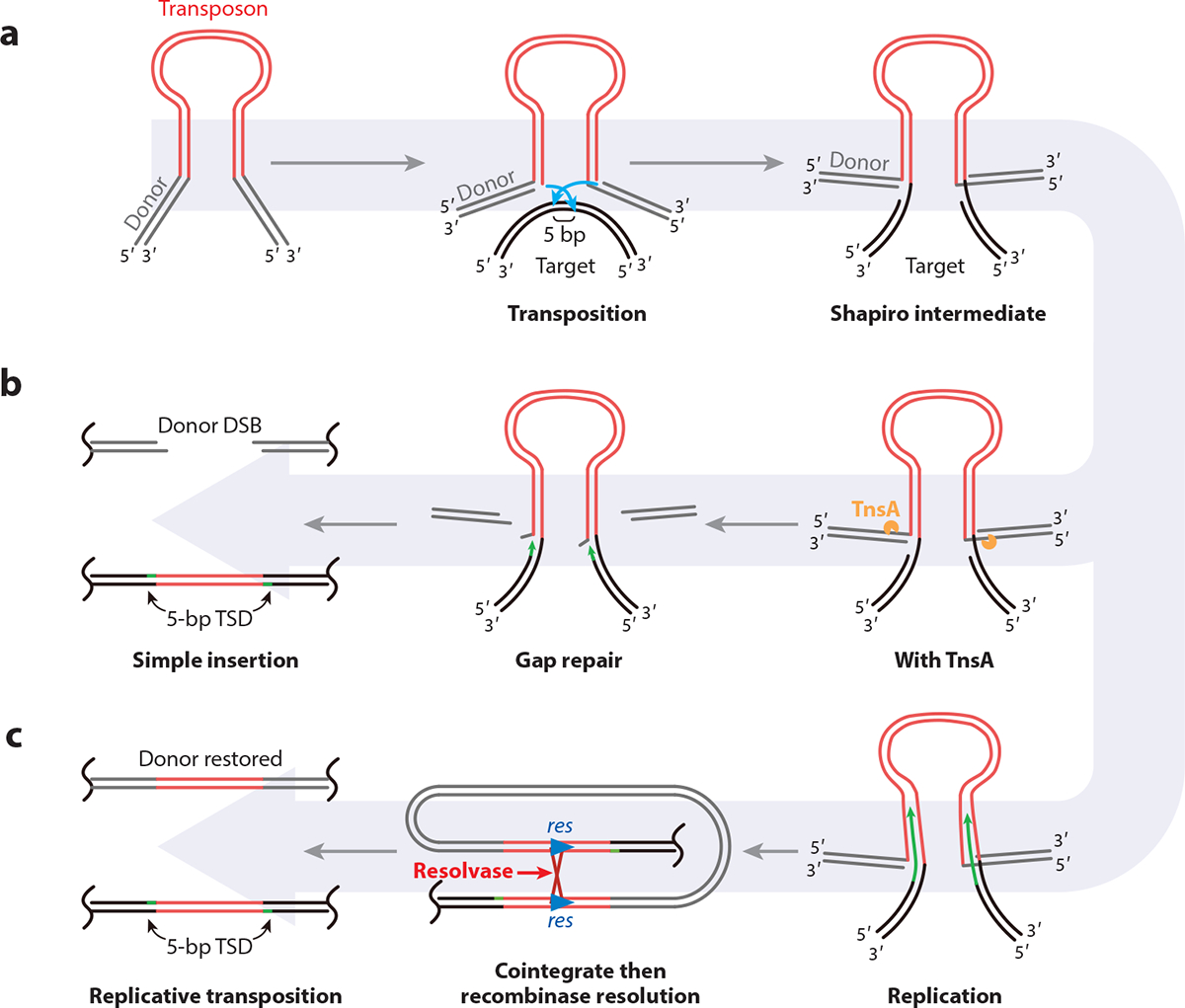
Tn7 transposition occurs via a cut-and-paste mechanism using a heteromeric TnsA+TnsB transposase. (*a*) TnsB makes breaks at the ends of the element that join to a target DNA in a coordinated fashion. The joining events in the target DNA are staggered by 5 bp. The activity of TnsB results in the formation of a so-called Shapiro intermediate structure. (*b*) TnsA can cleave the donor DNA flanking the transposon to allow the transposon to be integrated into the target as a simple insertion. The gaps flanking the transposon are repaired by host processes, leading to the 5-bp target-site duplication. (*c*) Transposons that lack TnsA can undergo a process known as replicative transposition. The free 3′ ends from the target DNA can be used to prime DNA replication that proceeds through the element, forming a cointegrate. A second recombination system can use site-specific recombination at a *res* site to allow resolution of the cointegrate, leaving an insertion in the donor and target DNAs. Host homologous recombination can also process the cointegrate, leaving an insertion in the donor and target DNAs (not shown). Abbreviations: DSB, double-strand break; TSD, target-site duplication.

All known CAST elements share certain basic features that were adapted from mobile DNA elements called DNA transposons, which were merged with diverse types of CRISPR–Cas bacterial defense systems. The transposon components allow the integration of a DNA fragment into a new location. To date, all the CAST elements are related to a special type of transposon called Tn7. Transposon Tn7 was the first transposon found to target a specific site on the *Escherichia coli* chromosome (5) and was later shown to guide targeting by recognizing a specific DNA sequence (6). Tn7 is capable of sequence-specific and nonsequence-specific transposition (5), attributed to five transposon-encoded gene products: TnsA, TnsB, TnsC, TnsD, and TnsE. The Tn7 transposase is heteromeric, formed by two proteins, TnsA and TnsB. Using two proteins allows transposition to occur by a cut-and-paste process where the element is completely excised from one location, leaving a DSB behind in the donor DNA (7, 8) (Figure 1). The TnsB transposase is a member of the large family of RNase-H fold proteins, which are also referred to as DDE proteins for the active-site residues used to coordinate the Mg^2+^ that is used in the active site of the protein. TnsB is responsible for making the breaks at the 3′ ends of the transposon in concert with a closely coordinated joining event to the target DNA. A DNA structure formed during this process that bridges the donor to the target DNA is known as the Shapiro intermediate (9) (Figure 1a). In the case of Tn7 the TnsA protein nicks the DNA just outside the 5′ ends of the element, completely freeing the element from the target DNA and allowing it to integrate into the donor DNA as a simple insert (8) (Figure 1b).

The transposon is joined at positions in the target DNA that are staggered by 5 bp, a process that leaves gaps on opposite strands flanking the element on each side (10, 11) (Figure 1). Repair of these gaps leads to the 5-bp target site duplication that is characteristic of Tn7 and all related elements. In addition to controlling the point of integration, Tn7 also controls the left-to-right orientation of the integrated DNA fragment. The process that allows orientation to be controlled is incompletely understood but seems to be found in all relatives of Tn7 (12–14) (see Section 2.6).

**Figure 2:**
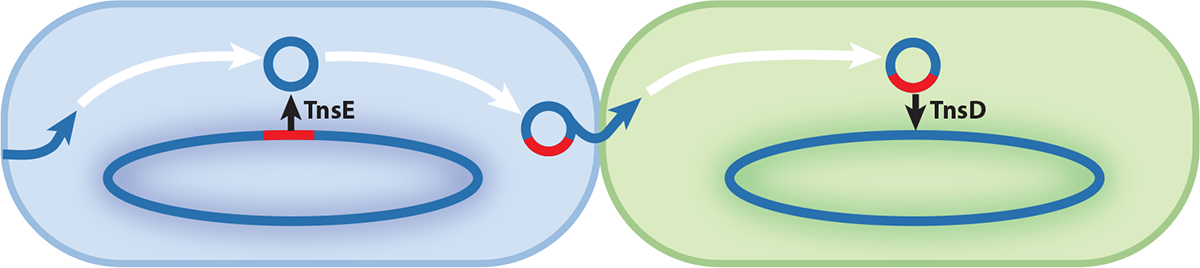
Illustration of the Tn7 two-pathway lifestyle. The Tn7 transposon is shown as a red line. Tn7 targets conjugal plasmids with target selector TnsE, facilitating horizontal transfer between bacteria. Once in a new host, the target-selector TnsD directs Tn7 transposition into the conserved *glmS* attachment site on the chromosome.

Tn7 transposition is directed in two transposition pathways by different target-selecting proteins, TnsD and TnsE. TnsD has a C-terminal DNA-binding domain that recognizes the conserved *glmS* gene 3′-end coding sequence (15). TnsD has an N-terminal TniQ domain that recruits transposon insertion through its interaction with the AAAC ATPase TnsC (16). TnsE recognizes the DNA replication–associated structure of lagging-strand synthesis, which allows Tn7 to preferentially target actively conjugating DNA (17–19). How TnsE recruits TnsC is not known. The two transposition pathways allow Tn7 to reside at a safe chromosomal site as a reservoir and to disseminate via conjugal plasmids. This tightly controlled two-part targeting strategy allows Tn7 to avoid the negative consequences of random transposition, which would run the risk of disrupting host genomic integrity, and maximizes dissemination (Figure 2). Transposase activity is latent until a target is captured by the target-site selecting proteins. Tn7 represents the first discovery from a vast group of transposons capable of selecting transposition targets; their great diversity and application potential has become increasingly obvious in recent years.

### 2.2. Diversity of Tn7-Like Transposons

There is currently no broadly accepted definition of a Tn7-like transposon due to their diversity and the observation that the elements do not show monophylogeny (20). Generally, the field considers transposons encoding TnsB and TnsC with the addition of TnsA and/or TniQ family proteins to be Tn7-like elements. A system for transposon naming has not been rigorously established. There is a transposon naming registry that is useful for cataloging transposons, but the sequential numbers assigned in this registry do not provide classification information that informs about their function (21) (Table 1). Certain notable transposons are often named after a particular representative that has been well studied. A parallel system of referring to transposons involves a letter code indicating the species of bacteria where they were initially found. For example, a well-studied CAST element that uses a type V-K CRISPR–Cas system from *Scytonema hofmanni* is commonly referred to as ShCAST (Table 1). A standardized naming system is needed, most likely similar to the systems used for naming restriction enzymes (22), integrating conjugative elements (ICE) (23), or retron elements (24), all of which involve characters representing the species name in which the system was first identified followed by a number. It would also be helpful to integrate more information about the phylogeny and interrelatedness of all Tn7-like elements into whatever system is adopted. The five known types of CAST are grouped by their independent cooption events from different types of CRISPR–Cas systems: type I-F3, V-K, I-D, and two examples from I-B (I-B1 and I-B2) CAST elements (25) (Table 1; see Section 3).

### 2.3. Two Complementary Pathways Maximize Dispersal of Tn7-Like Transposons

**Figure 3:**
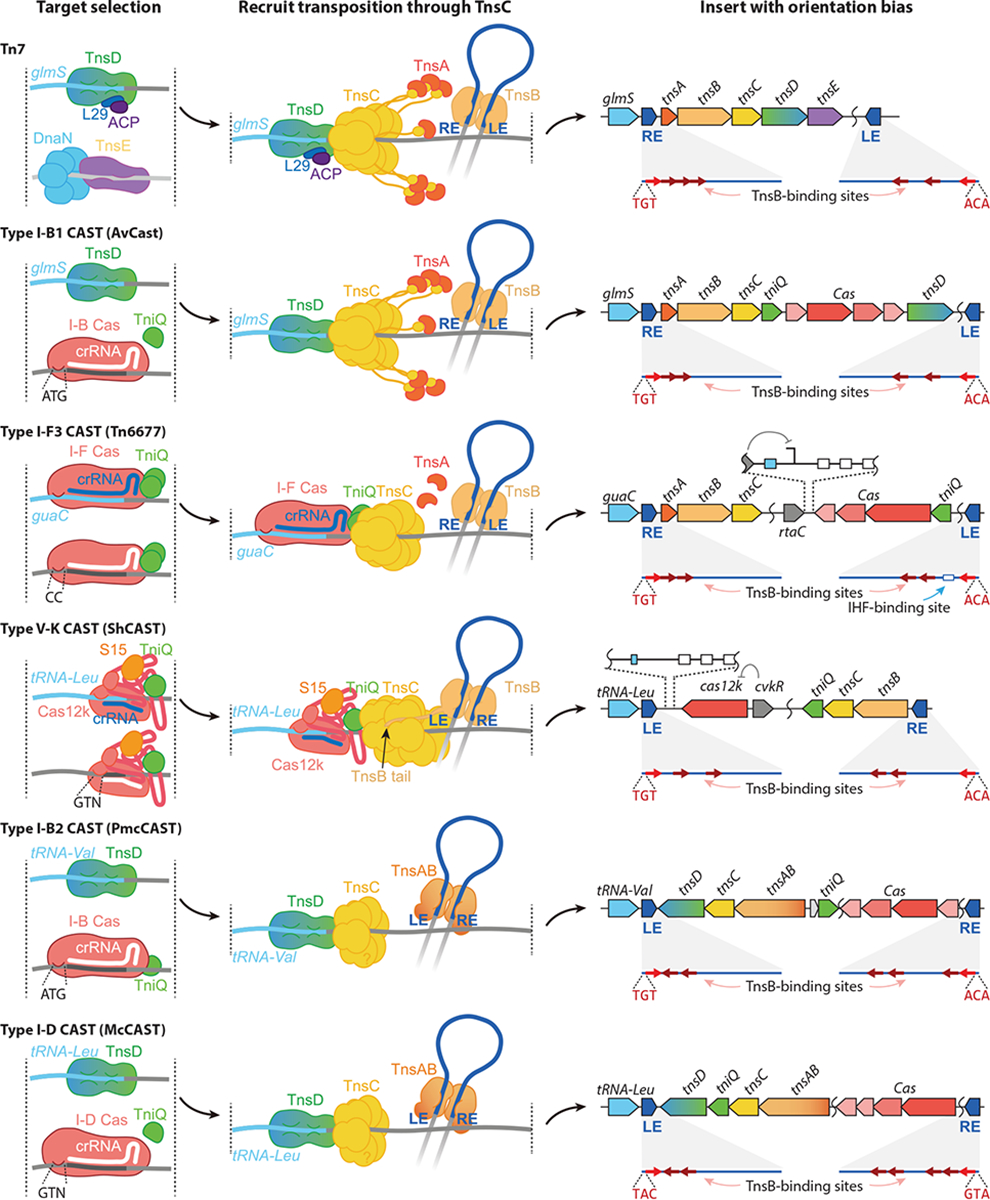
Tn7 and five CAST elements share important characteristics. Tn7 and known CAST elements convergently evolved dual transposition pathways. Type I-B1, I-B2, and I-D CASTs have two target-selecting TniQ domains, one has DNA-binding domains (TnsD) and another coopts Cas for RNA-guided transposition. Type I-F3 CASTs and type V-K CASTs have only CRISPR–Cas-guided transposition machinery, but they can use categorized spacers to achieve the dual transposition pathway. The coopted Cas genes all lost their adaptation and interference genes and functions. In addition, type V-K CASTs and type I-F3 CASTs also coopted transcriptional regulators for controlling RNA-guided transposition. Despite these convergences, different CAST families evolved independently from various branches of Tn7-like transposons, based on their differences in core transposition machinery. The transposon and CRISPR–Cas genes and the left and right ends are indicated. The sequences of the terminal 3 bp of the elements are indicated. For the CAST elements, the PAM sequences are indicated; the guide RNA is indicated and set in blue if it targets that attachment site or white if it is continuously programmed to target bacteriophages and conjugal plasmids. The CRISPR array is indicated with black diamonds for repeats and rectangles for the spacers. Abbreviations: ACP, acyl carrier protein; CAST, CRISPR-associated transposon; crRNA, CRISPR RNA; LE, left end; PAM, protospacer adjacent motif; RE, right end.

For transposons, transposition activity is likely evolutionarily constrained by the deleterious effects of random transposition. Target selectors help Tn7-like transposons minimize or essentially eliminate random transposition and its negative consequences. The inherent control of transposition activity opened the door to allow the evolution of numerous advantageous dissemination strategies. One near-universal strategy for Tn7-like elements is a two-pathway lifestyle. As mentioned earlier, two transposition pathways allow Tn7 to establish a reservoir in a stable chromosomal locus and to disseminate to other bacteria via other mobile elements, especially conjugal plasmids (Figure 2). Both pathways have evolved to occur at a high frequency, likely because the risk of host gene inactivation is the main evolutionary constraint to high-frequency transposition with most elements. All known CRISPR-guided transposons are believed to utilize a version of this strategy. Different Tn7-like elements coopted different CRISPR–Cas systems for targeting conjugal plasmids but use a variety of mechanisms to target transposition adjacent to a conserved chromosome gene (attachment sites or *att* sites). Attachment sites have also been called homing sites (26). Many CAST elements have two TniQ family proteins, one that communicates with a CRISPR–Cas system and another for targeting attachment sites (Figure 3; Table 1). For example, type I-B1 CAST elements, closely related to Tn7, also have a *glmS*-targeting TnsD (26, 27); type I-B2 CAST elements have a tRNA-targeting TnsD (26); and some type I-F3 CAST elements have a *parE*-targeting TnsD (28, 29). However, CAST elements do not necessarily need two target-selecting proteins to achieve the two-pathway lifestyle. All type V-K CASTs, most type I-F3 CASTs, and some type I-B1 CAST elements do not have additional TniQ/TnsD proteins for attachment-site targeting. Instead, in some cases, CAST elements can have guide RNAs that recognize the attachment site in the chromosome. As first revealed with type I-F3 CASTs, these elements categorize spacers for two transposition pathways (29). Similarly, type V-K CAST elements evolved the same strategy, with specialized spacers targeting various conserved genes, mostly tRNA genes (26, 30). One of the exciting characteristics of Tn7-like elements is that they repeatedly evolve the same strategy. For example, within the type I-B1 CASTs, there is a subgroup that independently obtained a CRISPR spacer to a safe chromosomal *att* site. This acquisition likely led to the subsequent loss of the TnsD responsible for *glmS*-targeting (31).

Despite the prevalence of the two-pathway lifestyle among Tn7-like elements, there are examples where it does not appear that two pathways exist. Most Tn5053 family transposons, for example, have only one TniQ protein and do not seem to have any other targeting pathways (Table 1). Tn5053 family transposons use their only TniQ to target the resolvase resolution sites associated with Tn1721 and closely related Tn3 family transposons, which are abundant on plasmids (32). Another example is the Tn5469 element (Table 1). Tn5469 has no cargo genes and has only one TniQ. This transposon was identified in a screen for spontaneous inactivation of a cyanobacterial gene, suggesting a lack of target specificity (33). Considering the diversity of TniQ proteins, more lifestyles are likely waiting to be discovered.

### 2.4. Target-Selecting Proteins

**Figure 4:**
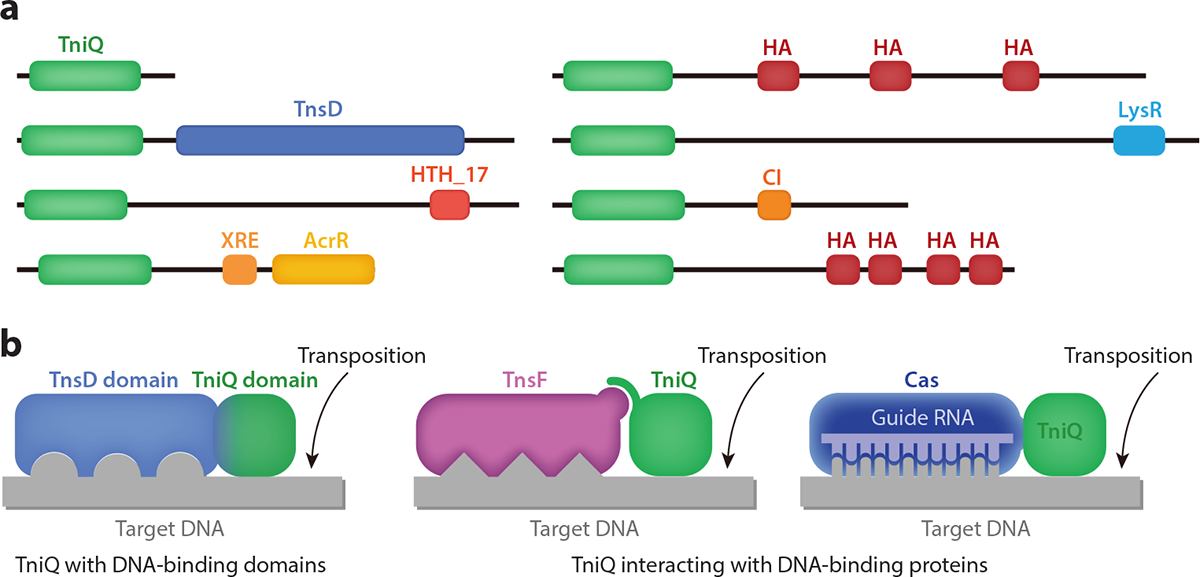
TniQ domain proteins function as highly modular adaptors to allow diverse modalities for targeting with Tn7-like elements. (*a*) Domain organizations of some TniQ proteins. Most TniQ domain–containing proteins have DNA-binding domains at C-terminal ends. (*b*) One major configuration involves the natural fusion to DNA-binding domains, like the TnsD domain. TniQ proteins can also noncovalently interact with CRISPR–Cas systems or with an accessory protein like TnsF. In all cases, transposition occurs at a point that is displaced from the sequence recognized. Abbreviations: AcrR, HTH-type transcriptional regulator AcrR; CI, phage CI repressor domain; HA, helicase-associated domain; HTH, helix-turn-helix domain; XRE, xenobiotic response element family HTH DNA-binding domain.

Tn7-like transposons have continually diversified, evolving novel transposition pathways over time. This likely underlies the importance of the need to continually move without insertional inactivation of essential or important genes and the need to move between cell populations. Incredible diversity exists in how the same four component proteins have adapted to allow differences in transposon lifestyle. TniQ domain–containing proteins are a near-universal component for Tn7-like elements. The TniQ domain interacts with a core transposition component, TnsC (see Section 2.5). TniQ proteins evolved to allow new targeting pathways by acquiring different kinds of DNA-binding domains or the capability to interact with CRISPR–Cas systems. In most cases, the ability of Tn7-like transposons to have two separate transposition pathways comes from having two separate TniQ proteins, one dedicated to each pathway (Figure 3). The TniQ domain appears to always form the N-terminal domain of these proteins, but the domain organization at the C terminal varies considerably (Figure 4), although it usually contains a DNA-binding domain, which presumably provides TniQ with DNA target specificity. Some minimal TniQ proteins without DNA-binding domains can also achieve target specificity by recruiting CRISPR–Cas (Figure 4), a functionality that has independently evolved multiple times (27). CRISPR–Cas-guided transposition represents an interesting but small fraction of the diversity of Tn7-like elements. A growing number of these systems have been investigated. As the underlying principles of the modularity of TniQ proteins are better understood, there should be a rich future for developing programmable genome engineering tools de novo.

The only known non-TniQ target-selecting protein family is TnsE. TnsE from prototypical Tn7 recognizes DNA features associated with lagging-strand DNA replication preferentially during the transfer of conjugal plasmids (17–19). Active conjugal DNA replication in recipient cells appears to provide a preferred target for the TnsE-mediated transposition pathway, but why this is the case remains mechanistically unclear (34). Features associated with replication termination and DNA DSB repair are also preferred targets for TnsE-mediated transposition, suggesting many nonstandard forms of DNA replication provide access to TnsE (35, 36). TnsE homologs are almost universal among *glmS*-targeting and *comM*-targeting Tn7-like elements, but there is no mechanistic information outside work done with TnsE from prototypic Tn7 (20, 37). In addition to TniQ and TnsE, other transposition modulators might also be target-site selectors. For example, TniM of Tn512 can increase the transposition rate when no proper target site is available (38). However, it is unclear whether TniM provides any degree of target specificity or selectivity.

### 2.5. Donor Transposon Recruitment Through TnsC

After target recognition, target-selecting proteins guide a transposition event into a position adjacent to the sequence recognized (Figure 3). This is achieved by recruiting the donor transposon with a diverse group of AAA+ proteins called TnsC proteins. TnsC provides the connection between the transposase proteins that interact with the ends of the transposons and a site recognized by the TniQ protein. Like many AAA+ proteins involved in DNA replication and recombination, TnsC proteins form oligomers on DNA (39–42). The detailed mechanisms and how they may differ between systems are still active areas of research. In all systems, the target-selecting proteins may lock a TnsC oligomer in place and change its conformation to recruit the transposon within a donor-DNA licensing transposition. While many TnsC AAA+ proteins are able to change the conformation of DNA, in the prototypic Tn7 system, TnsC is unable to alter the structure of the DNA. Multiple lines of investigation suggest that instead TnsC must load onto DNA that is distorted either by TnsD or in some other way (39, 43–45). For prototypic Tn7, a seven-member lock washer–like ring of TnsC that forms on DNA provides two important mechanistic advantages; it dictates that transposition occurs only on one side of the sequence that is recognized and also sets the distance between the sequence recognized and the point of insertion (39) (Figure 3). Studies on a type V-K CAST from ShCAST showed that its TniQ could cap the end of a TnsC filament, stabilize the DNA-bound TnsC, and thereby help define the fixed position for transposition (40, 41). The TniQ domain of Tn7 TnsD also likely interacts with its TnsC at the same interface as the ShCAST TniQ interacts with TnsC (39), suggesting the TniQ–TnsC interaction is a conserved mechanism for transposon recruitment and activation of transposition.

Interestingly, TnsC proteins from different Tn7-like transposons can vary considerably, supporting the idea that the different types of Tn7-like elements evolved independently and are regulated in different ways. For instance, the size of TnsC can vary from fewer than 300 amino acids in type V-K CAST to well over 500 amino acids in prototypic Tn7. They often also have different oligomeric states. ShCAST TnsC forms continuous helical filaments on DNA (40, 41), prototypic Tn7 TnsC assembles into heptameric open rings (39), and Tn6677 TnsC forms closed heptameric rings (42). Although all TnsC are AAA+ ATPase family proteins, mutation of the conserved Walker domains of these proteins can behave differently, further supporting the idea that there are mechanistic differences in how TnsC functions across Tn7-like elements. The Walker A and B motifs are conserved amino acid sequences required for ATP binding and hydrolysis. Mutating the catalytic glutamate residue in the Walker B motif of TnsC renders Tn7 hyperactive for transposition, even in the absence of target-site-selecting proteins that are normally required (39, 46); however, the same mutation severely compromised Tn6677 (42), the type V-K ShCAST (40, 41), and the type I-D McCAST (from *Myxacorys californica* WJT36–NPBG1) (31). Furthermore, multiple TnsC proteins of type V-K CAST elements do not naturally have canonical Walker motifs; some lack catalytic glutamate in their Walker B motif, suggesting they cannot hydrolyze ATP, and some do not have a typical Walker A motif, implying they may not even bind ATP. Future mechanistic work with how AAA+ proteins are harnessed for transposition will likely be informed by bioinformatics. For example, bacteriophage Mu integrates and replicates using a transposition mechanism that involves a transposase, MuA, that is guided for transposition by a AAA+ protein, MuB, and this process shows many similarities with the Tn7-like systems (47). However, the MuA (TnsB-like) and MuB (TnsC-like) system is highly diverged, branching separately from the Tn7-like elements, and presumably has important differences (37).

### 2.6. Integration and Orientation Control

#### 2.6.1. Tn7 and Tn7-like elements control the left-to-right orientation of integration.

According to convention, the transposon end that is upstream of the genes encoding the transposition proteins is referred to as the right end (Figure 3). Little is known about how orientation is controlled, but the ability to control the orientation of integration events opens exciting possibilities for gene editing. For the orientation of the ends of the element relative to the site recognized to be controlled, all the components involved must collaborate. The sequence recognized by a TniQ protein (with or without a partner component) must pass orientation information through an oligomer of TnsC, which must interface with a polarity-differentiating signal in TnsB. The TnsB protein that recognizes the transposon end must also distinguish between a right and a left transposon end. The transposon has an elaborate array of TnsB-binding sites at each of the *cis*-acting left and right ends of the element. The spacing of these TnsB-binding sites is important for differentiating the two ends. Early work with Tn7 indicated that one end is likely more important. In the case of prototypic Tn7, a synthetic element with two right ends is functional, but one with two left ends is not, indicating that the right end is somehow special in this element (13, 48). In the right end of the prototypic Tn7 element there are four partially overlapping 22-bp TnsB-binding sites, while TnsB-binding sites are spaced apart in the left end. A structure-based study of the right end from prototypic Tn7 indicated that the closely spaced TnsB-binding sites result in a bend in the DNA at the end, which may be important for its activity (14). It was shown that the final two TnsB-binding sites must overlap for transposon functionality. The *att* site sequence recognized by TnsD (TniQ) with prototypic Tn7 is also outside the right side of the eventual transposition integration event (Figure 3). Tn7-like elements seem to vary as to whether the *att* sequence recognized is on the left or right side of the element. Many Tn7-like elements appear to have one end with overlapping sites and one end with spaced sites. Whichever end has overlapping sites appears to be closest to the *att* site recognized by the TniQ domain protein, and this correlation may be important (Figure 3). However, end configuration can vary widely, and different mechanisms likely evolved among the diverse Tn7-like elements.

Transposon ends terminate with an 8-bp end sequence that typically ends with TGT/ACA and indicates to the most terminally bound TnsB proteins where to cut at the ends of the transposon (Figure 3). The 5′-TGT/ACA-3′ end sequence is also conserved in related non-Tn7-like DDE transposons, like IS481 family transposons. However, not all Tn7-like transposons have the same end sequence; some type I-F3 CASTs and the type I-D McCASTs have non-TGT ends (28, 31). The mechanistic explanation for this conservation is yet to be understood.

#### 2.6.2. Connections between the TnsB-bound ends and TnsC.

During the process of transposition, the TnsB-bound ends interact, forming a structure called a paired-end complex (PEC). Formation of a PEC is probably a conserved feature across all transposons. It is believed that the chemistry for breakage and joining occurs in *trans*, where the protomers bound to the left end cut at the right end and vice versa. A mimic of a transpososome complex immediately after integration has been assembled and visualized for the bacteriophage Mu transposition system and later for ShCAST, revealing interesting details of how the components interact while holding the ends together (49, 50). A disordered extension at the C terminus of TnsB was shown to interreact with a specific region of TnsCs in the filament, but how this interaction coordinates the recruitment process remains enigmatic (50). In the case of the ShCAST system, TnsB is believed to play an important role in modifying the TnsC filament to the appropriate length before activating transposition at a set distance between the sequence recognized by a guide RNA and the specific point of transposon insertion (40, 41, 50–52).

#### 2.6.3. The transpososome holo-complex.

The mechanism of transposon recruitment and activation of transposition is not fully understood for Tn7 or any of the families of Tn7-like elements. However, the breakage and joining events are believed to be coordinated in the transposition process. Once a break occurs at the end of the transposon, it is directly joined to a target DNA, a process that occurs in a transpososome complex that includes donor and target DNAs. Transpososome complexes have been assembled with the prototypic Tn7 system and studied biochemically (53, 54). A postintegration transpososome or holo-complex was visualized by cryo–electron microscopy for the type V-K CAST ShCAST system (51). A disordered C-terminal extension in TnsB ShCAST can attach to the TnsC filament, providing insight into transposon recruitment to the donor complex. A C-terminal extension of prototypic Tn7 TnsB and of the transposase from phage Mu likely plays the same role. However, the C-terminal peptide is not conserved across different Tn7-like transposons, and it is likely that multiple different mechanisms of complex assembly have evolved.

### 2.7. Differences Between Cut-and-Paste and Replicative Transposition

After the initial breaking and joining steps of transposition, Tn7-like transposons form the Shapiro intermediate (9), a structure that can become a cointegrate after processing by DNA replication (Figure 1). For example, for related transposons that lack TnsA, the structures within the Shapiro intermediate are recognized by a special host system that allows DNA replication to be initiated. DNA replication across the element converts the Shapiro intermediate into a cointegrate between the donor and target DNAs (55–57). Cointegration of donor and target DNA can result in unwanted genome rearrangements that would be damaging to the host. Diverse Tn7-like transposons have two primary mechanisms for handling cointegrates, either avoiding the formation of a cointegrate from the Shapiro intermediate or dealing with cointegrates after they form. One common mechanism that is also used by prototypic Tn7 involves the TnsA endonuclease cutting the transposons out of donor DNA, together with TnsB-catalyzed strand transfer, thereby preventing cointegrate formation and completely removing the element from the donor DNA (Figure 1b). A strategy used by other elements that lack TnsA involves processing the cointegrates using a separate type of DNA recombination system, a site-specific recombinase, like Tn5053 (Figure 1c; Table 1).

How the TnsA endonuclease is recruited to cut at the ends of the element differs between types of Tn7-like elements. In most cases with Tn7-like transposons, TnsA is a separate protein; however, there are multiple examples where the TnsA protein is fused with TnsB, like in type I-B2 CASTs and type I-D CASTs (Figure 3). Even when TnsA is a separate protein, TnsA recruitment mechanisms likely differ across transposons. In prototypic Tn7, the TnsC protein C-terminal domain can interact with TnsA, allowing TnsA to bind DNA (58), but this domain is missing in many Tn7-like transposons. Tn7-like transposons without TnsA are usually associated with a separate serine or tyrosine site-specific resolvase system (20). However, the type V-K CASTs have neither TnsA nor a separate site-specific recombinase system. A study on type V-K ShCAST confirmed the formation of cointegrates in *E. coli* (59). It is unknown whether type V-K CASTs have a way of dealing with cointegrates in their native cyanobacterial hosts. Many transposons outside of Tn7-like elements can also move by a cut-and-paste mechanism, but these typically involve a single kind of transposase that forms a hairpin on either the transposon or donor DNA during the reaction to remove the element from both DNA strands (60).

### 2.8. Target Immunity

Tn7 and some other types of transposons can strongly inhibit a second insertion from occurring in a region where the element is already found (61–63; R. DeBoy, N.L. Craig, unpublished observations). This property is important, because the DNA sequence recognized by TnsD (TniQ) or a CRISPR–Cas effector is offset from the point of integration and is maintained after transposition (Figure 3). The significance of this extends across Tn7-like elements, since cells transiently contain multiple copies of the chromosome during active cell replication. To safeguard genome stability, a mechanism is needed to prevent integration into a sister chromosome. The type I-F3 and V-K CAST systems seem to possess target immunity, although how the mechanism used for target immunity compares to the prototypic Tn7 system has not been examined (64, 65).

### 2.9. Cargo Genes of Tn7-Like Transposons

Tn7-like transposons often carry additional genes other than those directly related to transposition, called cargo genes (66). An analysis sampled Tn7-like transposons from 739 completely sequenced genomes and found that the most well-represented family of proteins was mobilome genes, followed by defense genes and genes for replication, recombination, and repair (20). Phage defense systems seem to be especially abundant in Tn7-like elements (28, 59). The abundant ISs (insertion sequences) found as cargo in Tn7-like transposons indicate that Tn7-like transposons likely frequently acquire new cargo genes in a process enabled by IS elements (20). Given that closely related Tn7-like elements can have vastly different cargo, it seems likely that there are undiscovered mechanisms for cargo capture.

## 3. DIVERSITY OF CRISPR–Cas TRANSPOSONS

### 3.1. Discovery of CRISPR–Cas Transposons

Prototypic Tn7 was studied for decades before it slowly became apparent that multiple types of Tn7-like elements existed (66–69). However, the true scope of this diversification was first appreciated with a comprehensive analysis indicating that 10–20% of bacteria had Tn7-like elements that fell into at least 22 different types based on TnsA phylogeny (27). This study also included bioinformatic support for a proposal that CRISPR–Cas systems carried by diverse Tn7-like elements were being used for guide RNA–directed transposition (27). Following bioinformatic identification of the first three examples of CRISPR–Cas cooption by transposons, two more were separately identified (31, 70), and currently, five independent cases of guide RNA–directed transposition have been verified experimentally in a heterologous host (26, 29, 31, 59, 64). Subsequent bioinformatic analyses suggest that there could be more that have yet to be confirmed experimentally (37, 71).

### 3.2. General Features of CRISPR–Cas Transposons

Canonical CRISPR–Cas systems function as adaptive immune systems in bacteria and archaea. They utilize a CRISPR RNA (crRNA) to recognize a target, which is subsequently either directly cleaved or degraded by loading a processive helicase/nuclease. This helps to inactivate bacteriophages and other mobile genetic elements (72–74). Cas proteins catalyze three stages: spacer acquisition (or adaptation), pre-crRNA processing, and interference. CRISPR–Cas systems are remarkably diverse, and they are categorized into two large classes, six types, and over 40 subtypes with some numeric subgroupings (25, 73). Class I systems are the most common, in which the functions in the effector complex are spread across a number of proteins (called cascade), while in Class II systems (e.g., Cas9), these functions are provided by one large protein (73).

Different types of Tn7-like elements have independently coopted diverse types of CRISPR–Cas systems (Figure 3). Based on the work performed thus far, the mechanism used in this cooption process seems to differ across the CAST systems, despite them all being mediated by TniQ family proteins. However, the coopted CRISPR–Cas systems share similar modifications; they have almost universally lost nuclease activity or genes required for processive DNA degradation, have lost spacer acquisition genes, and have shortened CRISPR arrays (27, 71). Type I-F3 CASTs and type V-K CASTs individually coopted transcriptional regulators from different sources to regulate RNA-guided transposition (29, 30, 75). The two-pathway lifestyle is another convergent feature shared by many Tn7-like transposons that was achieved through different approaches across CAST elements. It is also clear that some cooption events are more ancient and evolved, while others appear to be very recent.

### 3.3. Type I-F3 CRISPR-Associated Transposons

The type I-F3 CASTs are the largest group of CASTs in sequenced genomes (27, 76). The majority of the I-F3 CASTs are found in Vibrionaceae (77), but this CAST group extends somewhat broadly across gammaproteobacteria and is the only known group of CAST elements outside of cyanobacteria. Most type I-F3 CASTs have separate TnsA and TnsB proteins, but they are functional when fused and are sporadically found naturally fused in some elements (29, 78). The TnsA protein from type I-F3 CASTs is most like another group of Tn7-like elements, Tn6022 elements (however TnsA was coopted separately in this group) (20, 27, 76). Tn6022 elements play a central role in the acquisition of antibiotic resistance in the pathogen *Acinetobacter baumannii* (79). Type I-F3 cascade was coopted from the well-studied canonical type I-F1 CRISPR–Cas systems, but little is known about the type I-F3 CAST transposition machinery. Work in this system first showed that TniQ formed an important interface between the effector and the rest of the transposition proteins (59, 80).

#### 3.3.1. Type I-F3 CRISPR-associated transposons use CRISPR RNAs for two transposition pathways.

Work with type I-F3 CASTs indicates that a dedicated crRNA is used to direct transposition into a chromosomal *att* site, and different guides encoded in a CRISPR array are mostly used to recognize mobile plasmids (29). Encoding a crRNA that recognizes the host chromosome would be detrimental to the host when the element coexists with canonical type I-F1 systems. This is because the guide RNA that recognizes the host chromosome could lead to degradation of the host chromosome by canonical type I-F1 systems. Work with the I-F3 CASTs revealed that they have multiple strategies to prevent other CRISPR–Cas systems from using the transposon-encoded crRNAs (29, 81).

#### 3.3.2. Type I-F3 CRISPR-associated transposons fall into diverged subfamilies.

The type I-F3 CAST elements fall into two major branches, the I-F3a and I-F3b elements, which regulate transposition differently using independently coopted Xre family proteins, called RtaC and RtbC (29) (Figure 3). Both branches of I-F3 elements show that zygotic induction allows a burst of transposition when they enter a new cell. The I-F3a elements express only a crRNA that recognizes an attachment site upon entering a new host, which would limit the opportunity for a canonical I-F1 system to use this crRNA to degrade the host chromosome. Type I-F3b elements were also found to categorize crRNAs by encoding normal and specialized atypical crRNAs that are highly diverged in their repeats. Atypical crRNAs are hyperactive for RNA-guided transposition and can only be used efficiently by type I-F3 CASTs (not canonical I-F1 systems) (29). The process that allows crRNAs to be categorized is controlled by TniQ, which forms an intimate interaction with the effector complex and the crRNA (81). TniQ was also found to be required for the formation of a complete R-loop with the crRNA, which is likely a key licensing step before recruitment of TnsC and then the transposase. Type I-F3 CRISPR–Cas systems also show other differences from the canonical I-F1 systems, namely protospacer adjacent motif (PAM) ambiguity and mismatch tolerance (29, 81). The crRNAs used to recognize the chromosome *att* sites are mostly limited to four different *att* sites across type I-F3 CASTs, the *guaC* and *yciA* genes (type I-F3a elements) and the *ffs* and *rsmJ* genes (type I-F3b elements). PAM ambiguity and mismatch tolerance are likely beneficial because they allow the type I-F3 effector complex to recognize the *att* sites found in different bacteria even when the sequence diverges (for example, due to variation in the wobble position with different codon biases). PAM ambiguity and mismatch tolerance were also found to help keep the crRNA private to the transposon because a canonical I-F1 system lacked this tolerance for changes in the PAMs and mismatches (29, 81). As mentioned in Section 2.3, a third branch of type I-F3 CAST elements uses two TniQ proteins to allow two pathways; in these elements, the attachment site in the chromosome is directly recognized by one TniQ that directs insertion adjacent to the *parE* gene, and a second TniQ that interfaces with the CRISPR–Cas system is used for directing transposition into mobile elements capable of cell-to-cell transport of the transposon.

#### 3.3.3. Diverse type I-F3 CRISPR-associated transposons and opportunities for genome editing.

Since their original discovery many diverse elements from the type I-F3 CASTs have been established outside of their native hosts (28, 29, 82, 83). Having multiple functional orthogonal elements should help support the development of gene editing tools in diverse species. Divergence is already proving useful by allowing multiplexing for working with multiple systems in a single host (28, 65). Having multiple orthologs has also helped obtain the first proof of principle showing the system can function in human cells (84). Evidence for a role of host factors with the I-F3 system may contribute to adapting the I-F3 system for high-frequency editing (85). For example, some type I-F3 elements are dependent on a bacterial host DNA-binding protein, IHF, while other orthologs are not.

In a heterologous *E. coli* host, where most of the work has been done, on-site targeting differs between crRNAs and the specific site chosen but is generally over 95% (29, 65, 78, 85). Comparing insertion patterns across different crRNAs has also found important biases that should help with designing the most precise targeting. The choice of crRNA plays the largest role in determining integration efficiency; however, base-pair contacts at the point of integration can also help determine how much precision is found with integration (85). Integration efficiency varies with the assay but can approach 100% by some measures. At high efficiency, target immunity can become more important. Initial work suggests that these systems should be useful for future applications involving precision editing of genes within complex microbiome populations (86). The size of the DNA fragment that can be integrated with these systems is likely to be large because these elements naturally can be many tens of thousands of base pairs in size. The major complication for the editing prospects of I-F3 CAST elements is the number of components that are needed.

### 3.4. Type V-K CRISPR-Associated Transposons

The type V-K CASTs are the second largest group of CASTs in sequenced genomes and are natively found only in cyanobacteria (30, 70). Type V-K CAST systems use the single effector Cas12k protein and are the only non–type I systems that have been discovered and characterized (27, 64). Three different type V-K CASTs were shown to be functional in a heterologous *E. coli* host (64, 65) (Table 1). Most work has been done with the ShCAST system, which has been reconstituted in a robust in vitro system (64) and has been amenable to structural studies (40, 41, 50–52, 87). Excitingly, ShCAST shows some functionality in human cells (88). There are also multiple drawbacks with the type V-K CASTs. Transposition seems to occur with a much higher level of off-site targeting than with the other CAST systems (78). This contrasts with targeting in the natural setting, as in natural cyanobacterial genomes, nearly all of the insertions can be explained by an *att* site–targeting crRNA encoded in the element (30). There could be an undiscovered specificity host factor in the native host or spacers may somehow be naturally optimized in the native setting, among other explanations. The type V-K systems also do not encode the TnsA endonuclease and transpose using a replicative mechanism, which complicates their use in most applications due to the formation of cointegrates (78, 90, 91) (Figure 3). Attempts have been made to impart a TnsA-like activity to help mitigate this significant drawback (88).

#### 3.4.1. Type V-K systems are the only known CRISPR-associated transposons that use a single effector protein.

Unlike the other CAST systems, there is no evidence of an extant canonical type V-K CRISPR–Cas system. The type V Cas12 effectors are remarkably diverse, likely because they evolved from an especially diverse family of proteins, the TnpB proteins (89, 92). It is possible that the type V-K systems evolved directly from a TnpB protein and not an ancestral canonical type V system. TnpB proteins are nucleases, and Cas12k proteins were found to have a natural loss-of-activity mutation, which prevents the system from acting like a canonical CRISPR–Cas defense system (70). Like other CAST elements, the type V-K systems do not encode their own adaptation systems and must rely on spacer acquisition in *trans* when they reside in a host with an adaptation system from other CRISPR–Cas systems. Given the nature of the arrays found with these elements, it has been suggested that a canonical type I-D adaptation system may be used for adaptation with type V-K CASTs (30). However, these guides are not usable for interference by canonical type I-D systems, which explains how type I-D CASTs can coexist in the same cell as canonical I-D CRISPR–Cas systems without autoimmunity (30).

#### 3.4.2. Type V-K CRISPR-associated transposons use CRISPR RNAs for two transposition pathways.

Like the type I-F3 CASTs, the type V-K elements categorize crRNAs within the element (26, 30). In nearly all cases the crRNA that recognizes the *att* site used by the element is truncated and in a separate position from the other crRNAs. Most of the type V-K CAST *att* sites are diverse tRNA genes (30, 70). Genes for tRNAs are common integration sites for many kinds of mobile elements that have fixed integration sites, including bacteriophages, diverse Tn7-like elements, and ICEs. Interestingly, across Tn7-like elements, different tRNA genes have been repeatedly acquired as *att* sites and by different mechanisms, namely by CRISPR–Cas-based mechanisms and through TnsD and TniQ proteins (26, 30, 31). For unknown reasons, *att* site usage is more dynamic with type V-K elements than with the type I-F3 system. While four *att* sites are typically used across the diverse type I-F3 CASTs, even within closely related type V-K CASTs, the *att* sites can differ (29, 30).

#### 3.4.3. The minimal type V-K CRISPR-associated transposons have exciting possibilities for reengineering.

The type V-K CAST system generally uses fewer components and smaller proteins than the other CAST systems (Figure 3). These attributes have contributed to success with mechanistic and structural work, including a holoenzyme structure of the complex with the Cas12k effector protein and all the transposon components poised on a DNA mimic to show integration (51). The small size and high activity of the type V-K CASTs suggest that they should be amenable to rational design as the basic mechanistic understanding advances. In addition to the limitations mentioned above, i.e., high levels of off-site targeting and moving by replicative transposition, the best-studied type V-K CAST has also been shown to have host-factor requirements. In the ShCAST system, assembly of the Cas12k–TniQ complex occurs with the host ribosomal protein S15 (93), which may limit its utility outside bacteria without reengineering. Interestingly, the prototypic Tn7 target-selecting protein TnsD (TniQ) has also coopted a host ribosomal protein, L29, and uses the acyl carrier protein (ACP) to assist in the formation of the target complex at its integration site (94).

### 3.5. Type I-B1 CRISPR-Associated Transposons

Type I-B CRISPR–Cas systems were independently coopted by very different types of Tn7-like elements (27). The type I-B1 CAST is a close relative to the prototypic Tn7 element. It uses two TniQ domain proteins, one of which is TnsD, which targets the conserved *glmS* site just like Tn7, and the other is TniQ, which coopted the type I-B CRISPR–Cas for RNA-guided transposition (26, 27) (Figure 3). This system has been naturally found only in cyanobacteria. The type I-B1 AvCAST system was established in vivo in *E. coli* and in a reconstituted in vitro system (26).

The I-B1 group shows multiple examples of convergent evolution across the Tn7-like elements. For one, it acquired the *comM* gene as an *att* site, like the Tn6022 element, but in this case, it acquired a crRNA that recognizes the coding sequence of the gene (31, 67). This also represents a case where a CAST element evolved to use a single TniQ protein that interfaces with a CRISPR–Cas system, similar to what is found with the type I-F3 and V-K systems (26, 29–31) (Figure 3). Second, CRISPR–Cas coopting TniQ in the I-B1 group appears to have evolved from a truncated TnsD, retaining a partial TnsD domain at its C terminal (26).

The type I-B CAST systems lack their own spacer acquisition system (adaptation genes Cas1 and Cas2) and presumably acquire spacers in *trans* from canonical type I-B CRISPR–Cas systems. It is unclear if crRNA within the CAST element can be utilized by canonical I-B CRISPR–Cas systems or if they have a mechanism for privatizing them to the transposon. The mechanistic details of how the transposon proteins and type I-B1 cascade interface has not been investigated.

### 3.6. Type I-B2 CRISPR-Associated Transposons

Very different types of Tn7-like transposons separately recruited type I-B CRISPR–Cas systems for guide RNA–directed transposition (27). In the case of the type I-B2 CAST, the Tn7-like family is from a group where the TnsA and TnsB proteins are fused as a single protein (Figure 3). This group of transposons identified within the cyanobacteria exemplifies the modular nature of Tn7-like elements (31). Elements that are closely related by the phylogeny of the TnsA–TnsB fused protein can be found with the type I-B2 system and the I-D system described in Section 3.7, as well as various other modalities for targeting that do not involve CRISPR–Cas systems. This suggests that important insights into cooption should be gleaned from these transposition systems. Having naturally fused TnsA and TnsB partially simplifies moving the system to heterologous hosts.

A structure of the I-B2 CAST system indicates that a different mechanism is used for the transposon components to interface with the CRISPR–Cas components than that used by I-F3 CASTs. While TniQ interacts with Cas6 in the I-F3 CAST element, in the I-B2 system, TniQ interreacts with Cas11, Cas7, and Cas6 (95). Like the other type I-B CASTs, the type I-B2 system must acquire a spacer in *trans*, likely from canonical I-B CRISPR–Cas systems.

### 3.7. Type I-D CRISPR-Associated Transposons

The type I-D and I-B2 CASTs evolved from the same subfamily of Tn7-like elements that have fused TnsA and TnsB proteins and use a TnsD (TniQ) protein that evolved to use different tRNA genes as attachment sites (31) (Figure 3). Within the branch of Tn7-like elements there is a notable amount of promiscuity in the exchange of TniQ partners. In addition to the type I-D and I-B2 cascade systems, there is a variety of other unstudied non-CRISPR–Cas targeting systems (31). It seems likely that understanding the mechanistic flexibility within this family of Tn7-like elements will provide important details about the cooption process. The I-D CAST from *Myxacorys californica* WJT36–NPBG1 (McCAST) was established as a programmable guide RNA–directed element in a heterologous *E. coli* host (31). The McCAST element displays efficient targeting and the expected strict orientation bias that is a hallmark of Tn7-like elements (29, 59, 65, 78). Given the nature of the canonical type I-D CRISPR–Cas systems, cooption for transposition also shows differences from the other type I systems. For example, the type I-D CAST system shows natural inactivation of the Cas10 nuclease by an active-site mutation (31) (Figure 3).

The I-D CAST system demonstrated flexibility in guide RNA length and could be engineered to function with ribozyme-based self-processing guide RNAs, removing the requirement for one cascade component (i.e., Cas6). The cascade protein Cas6 is known to form a critical interface with TniQ from the transposition machinery in type I-F3 CASTs (80, 96, 97). However, Cas6 was dispensable for guide RNA–targeted transposition for the I-D CAST, indicating a novel, unknown mechanism is used (31). Even though the transposition components are highly similar (~50% amino acid identity), the cascade functions used for target selection with type I-B2 and I-D CASTs are highly diverged (31). This surprising finding, that highly similar transposition systems are able to coopt diverse CRISPR–Cas systems, should provide important information about conserved features that can be used for cooption.

There are very few examples of type I-D CASTs, and they are all found in cyanobacteria. Multiple attributes of these elements suggest that cooption is much more recent than in the other CAST systems. For example, PAM usage is conserved and the core cascade components are more similar to their counterparts in the canonical systems than the other CASTs. Excitingly, an apparent middle step between life as a canonical CRISPR–Cas system and one used for guide RNA–directed transposition was identified (31, 37) and also established in a nonnative *E. coli* host (37).

## 4. OUTLOOK

**Figure 5:**
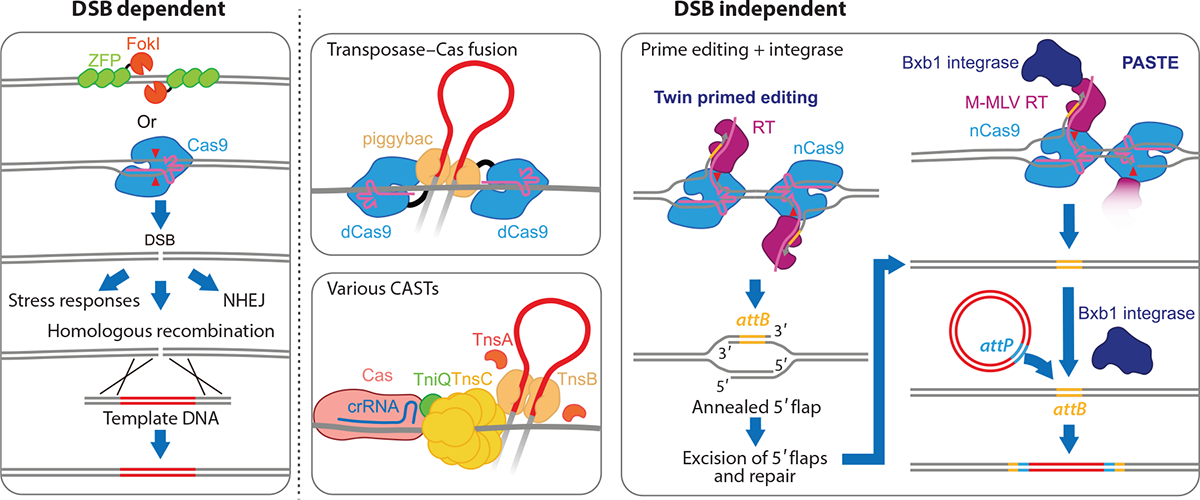
Early strategies for integrating large DNA sequences into genomes involved homologous recombination of a template DNA integrating as a repair template at programmed DNA breaks targeted with ZFPs or Cas9. Limitations of the DSB-dependent systems included the need to bias homologous recombination from direct joining by NHEJ and the stress responses associated with the DNA-damage response. Newer systems that utilize integrases and transposases show great promise for integrating large DNA sequences without the need for a DSB. Prime editing techniques can integrate the specific sequence (*attB*) needed for a cognate site-specific integration system to allow DNA cargo integration using recombinases like Bxb1 integrase. Abbreviations: CAST, CRISPR-associated transposon; CRISPR, clustered regularly interspaced short palindromic repeats; crRNA, CRISPR RNA; dCas9, endonuclease deficient Cas9; DSB, double-strand break; nCas9, Cas9 nickase; M-MLV, Moloney murine leukemia virus; NHEJ, nonhomologous end joining; PASTE, programmable addition via site-specific targeting elements; RT, reverse transcriptase; ZFP, zinc finger protein.

There has been brisk progress in the development of gene-editing tools over the last decade to address the limitations of systems that rely on host HR at targeted DSBs (Figure 5). While de novo engineered strategies coupling transposases to CRISPR–Cas systems met with limited success, diverse Tn7-like systems that have naturally coopted CRISPR–Cas systems offer considerable promise for integrating large DNA sequences in a programmable way. The ability to avoid inducing a DSB in the target DNA is also being engineered into additional systems derived from mobile DNA elements. In some of the exciting systems being developed, prime editing strategies that are suited for small editing procedures are being used to introduce site-specific integration sites (*attB*) for various integrases in procedures know as twin prime editing and PASTE (programmable addition via site-specific targeting elements) (98, 99) (Figure 5). An exciting future lies ahead on the final frontier of gene editing with the development of tools suited for integrating large DNA sequences into target genomes with high precision.

## 5. SUMMARY AND PERSPECTIVE

Naturally occurring guide RNA–directed transposons that have coopted diverse types of CRISPR–Cas systems provide an exciting example of how molecular systems are continually repurposed over time. Tn7-like transposons indicate how exquisite control over transposition activity provides an evolutionary starting point for diverse target-capture systems. In the short time since their discovery, work from many labs has already shown their potential in nonnative hosts. The initial evidence for functionality in human cells is particularly exciting, although vast increases in activity are needed for future work in human therapeutic gene editing. Additionally, as with many gene-editing technologies, gene-delivery systems remain a challenge due to the number of components that are needed to reach their medical potential (100–103). In the case of CAST systems, engineering prospects will benefit from the broad diversity of systems found in bacteria. Understanding the basic features of all CAST systems will bring the field closer to the aspirational goal of setting up a system where associations between a transposon and any CRISPR–Cas system could be engineered de novo. The appeal of this idea is that other type I CRISPR–Cas systems that are already functional in human cells and model eukaryotic systems could be adapted directly as CAST systems.

## Figures and Tables

**Table 1:** Examples of Tn7-like elements

Tn number	Other names	Features
Tn7	None	Targets *glmS att* site with TnsD (TniQ) and conjugal plasmids with TnsE (68)
Tn6230	None	Targets *yhiN att* site, but molecular details are unknown (67, 69)
Tn6022	AbaR	Targets *comM att* site to inactivate the gene with TniQ+Orf3 and conjugal plasmids, presumably with TnsE (67)
Tn5053	None	Targets plasmid resolution sites, presumably with TniQ
Tn6677	VchCAST	Type I-F3a CAST (59)
Tn6900	AsCAST	Type I-F3b CAST (29)
Tn7017	EasCAST	Type I-F3 CAST with a TniQ-targeted *parE att* site (28)
Tn7743	AvCAST	Type I-B1 CAST with a TniQ-targeted *glmS att* site (26)
Tn7744	PmcCAST	Type I-B2 CAST with a TniQ-targeted tRNA gene *att* site (26)
Tn7575	McCAST	Type I-D CAST with a TniQ-targeted tRNA gene *att* site (31)
Tn7745	CyCAST	Type I-D CAST with a predicted active CRISPR–Cas system (37)
Tn7747	ShCAST	Type V-K CAST (64)
Tn7746	AcCAST	Type V-K CAST (64)
Tn6999	ShoINT	Type V-K CAST (65)
Tn5469	None	Appears to have only untargeted transposition (31)
